# Unraveling the Interaction between FcRn and Albumin: Opportunities for Design of Albumin-Based Therapeutics

**DOI:** 10.3389/fimmu.2014.00682

**Published:** 2015-01-26

**Authors:** Kine Marita Knudsen Sand, Malin Bern, Jeannette Nilsen, Hanna Theodora Noordzij, Inger Sandlie, Jan Terje Andersen

**Affiliations:** ^1^Department of Biosciences, Centre for Immune Regulation (CIR), University of Oslo, Oslo, Norway; ^2^Department of Immunology, Centre for Immune Regulation (CIR), Oslo University Hospital Rikshospitalet, Oslo, Norway; ^3^Institute of Clinical Medicine, University of Oslo, Oslo, Norway

**Keywords:** albumin, FcRn, albumin-based therapeutics, IgG, half-life, recycling, transcytosis

## Abstract

The neonatal Fc receptor (FcRn) was first found to be responsible for transporting antibodies of the immunoglobulin G (IgG) class from the mother to the fetus or neonate as well as for protecting IgG from intracellular catabolism. However, it has now become apparent that the same receptor also binds albumin and plays a fundamental role in homeostatic regulation of both IgG and albumin, as FcRn is expressed in many different cell types and organs at diverse body sites. Thus, to gain a complete understanding of the biological function of each ligand, and also their distribution in the body, an in-depth characterization of how FcRn binds and regulates the transport of both ligands is necessary. Importantly, such knowledge is also relevant when developing new drugs, as IgG and albumin are increasingly utilized in therapy. This review discusses our current structural and biological understanding of the relationship between FcRn and its ligands, with a particular focus on albumin and design of albumin-based therapeutics.

## Introduction

Albumin and immunoglobulin G (IgG) are the two most abundant circulating proteins in the bloodstream, and account for an incredible 80–90% of the total protein pool. In addition, both share an extraordinary long serum half-life, which in humans amount to 19–21 days (1–3). While IgG is solely produced by B and plasma cells in response to foreign substances, such as pathogens, and is absolutely fundamental for protection against infections, albumin is produced by hepatocytes of the liver and acts as a multi-carrier of a plethora of insoluble and hydrophobic endogenous and exogenous ligands, such as ions, fatty acids, amino acids, and waste products as well as a range of biomedical drugs. In addition, albumin maintains the osmotic blood pressure, is an important antioxidant, and possesses enzymatic properties (4–6).

Despite their completely unrelated structures and biological roles, IgG and albumin share two common and unique features; extended serum half-life and an inverse relationship between serum concentrations and half-life (1–3). These characteristics were initially proposed to be caused by the presence of specific saturable receptor-mediated mechanisms that would protect the proteins from intracellular degradation (7, 8). And indeed, it later became apparent that a broadly expressed cellular receptor, then named the neonatal Fc receptor (FcRn), does exist and is responsible for salvaging both IgG and albumin from cellular catabolism via strictly pH-dependent recycling and transcytosis pathways. Thus, FcRn plays a key role in homeostatic regulation of these unrelated soluble proteins, securing a broad biodistribution throughout the body of both molecules (9–13).

Due to its well-recognized serum stability and longevity, albumin has been utilized as a carrier for drugs for a long time, either by direct genetic fusion or conjugation, or by non-covalent association of the drug to albumin (14–16). However, during the development of these strategies, the importance of the FcRn-dependent mechanisms for drug pharmacokinetics and pharmacodynamics was not really taken into account, as they were established before the FcRn–albumin relationship was appreciated. Now, care must be taken that the FcRn interaction is not disrupted when albumin-based therapeutics are designed and evaluated. In addition, we have shown that there are large differences in cross-species binding characteristics of human albumin to mouse and rat FcRn that compromise preclinical *in vivo* evaluations in rodents (17, 18). Thus, the pharmacokinetics and pharmacodynamics of a numerous human albumin-based therapeutics may have to be reassessed, and their FcRn binding ability and transport properties at different body sites taken into account. Furthermore, unmasking the molecular interaction of FcRn with albumin has given rise to new classes of engineered albumin variants with altered FcRn binding and transport capacities. Last, but not least, FcRn is well known to mediate transport of IgG across cellular barriers such as polarized epithelial cells covering mucosal surfaces and the placenta (19–24), which have been successfully utilized as gateways for oral, nasal, pulmonary, and *in utero* delivery of IgG-based therapeutics and vaccines (25–32). Whether albumin can be efficiently shuttled by FcRn via these pathways has not yet been fully addressed and needs to be explored, as such routes may be attractive for delivery of albumin-based therapeutics. In this review, we describe the current molecular and cellular understanding of FcRn and its relationship with its ligands with a particular focus on albumin biology and design of albumin-based therapeutics.

## The History of FcRn

F.W. Rogers Brambell (1901–1970) was the first to postulate the presence of a cellular receptor responsible for active prenatal transport of IgG from the mother to the fetus across the yolk sac in rabbits and across the intestine of neonatal rats (7, 33). This was based on experiments done in his own laboratory as well as work done by others, which showed that maternal IgG derived from mother’s milk was absorbed from the gut lumen of neonatal rats for up to 18–21 days post birth before the transport rapidly ceased (34). Also, using *in vitro* intestinal cell assays, it was shown that the transport was highly selective for the IgG isotype and solely dependent on the constant Fc part (35). Based on these observations, Brambell proposed that a single cellular receptor was the key player in mediating transcytosis of IgG over these cellular barriers (7).

Furthermore, Fahey and Robinson demonstrated that IgG was eliminated from the blood circulation in a concentration-dependent manner, as injection of high doses of IgG in mice greatly accelerated the clearance of endogenous IgG, while excess amounts of IgA, IgM, or serum albumin did not (36). Again, the process was fully dependent on Fc. Brambell recognized the resemblance between this work and his own studies, and proposed that a common receptor was responsible for transepithelial and materno-fetal transport, as well as for serum half-life regulation (7, 37).

During the 1970s, it was shown that transport of IgG across the intestinal epithelium of the neonatal rat was strictly pH dependent, as IgG uptake from the mother’s milk for delivery to the offspring would only happen at acidic intestinal pH and not at physiological pH (38). Almost 10 years later, the receptor in question was identified in tissue from the neonatal rodent gut as a heterodimeric protein consisting of 40–46 and 12 kDa subunits (39). This was followed up by cloning of the corresponding genes, which revealed that the 12 kDa subunit was β_2_-microglobulin (β_2_m) while the larger subunit was a heavy chain (HC) related to the major histocompatibility complex (MHC) class I (40). The discovery inspired its name FcRn.

As a parallel to postnatal transport across the neonatal intestine and the prenatal transport of IgG across the yolk sac of rabbits, a human ortholog of FcRn was cloned from syncytiotrophoblasts of the human placenta by Story and colleagues (41), and shown to direct transcytosis of mothers IgG to the fetus during the third trimester of pregnancy (24). In all cases, FcRn-mediated transcytosis ensures transfer of passive immunity to the fetus and the newborn. However, FcRn function is not restricted to neonatal life, as a large body of evidence has shown that it is expressed in a range of cell types in all species studied throughout life (20, 21, 23, 30, 42–53).

In contrast to the well characterized FcRn–IgG relationship, an explanation for the long half-life of albumin was missing for decades. Brambell did only briefly touch upon albumin, but did not discuss its catabolic rate (54). Instead, during the 1960s, Schultze and Heremans postulated that the proposed mechanism for IgG protection from degradation could also explain the correlation between the half-life and concentration-dependent catabolic rate of albumin (8). This was based on studies of the relative catabolic rates of IgG and albumin in patients suffering from agammaglobulinemia and analbuminemia. The work was largely ignored until Anderson and co-workers reported that FcRn binds albumin (11). The interaction was identified by chance when bovine albumin was co-eluted with recombinant soluble human FcRn from an IgG affinity column (11, 55). Like the FcRn–IgG interaction, the FcRn–albumin interaction is remarkably pH dependent (11, 56, 57). Whereas it was already known that mice, which do not express a functional FcRn, catabolize IgG more rapidly than normal mice (9, 10, 58), the same rapid catabolic rate was now shown also for albumin (11).

## The Architecture of FcRn

**Figure 1:**
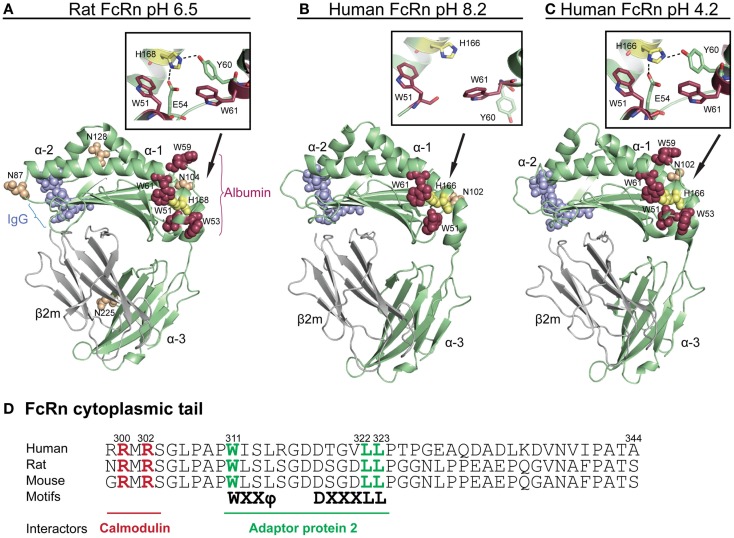
**Crystallographic illustrations of rat and human FcRn**. Crystal structures of truncated **(A)** rat FcRn solved at pH 6.5, **(B)** human FcRn solved at pH 8.2, and **(C)** pH 4.2. The FcRn HC is shown in green and the β2m subunit in gray **(A–C)**. The three domains of FcRn are denoted as α1, α2, and α3. The key amino acid residues involved in binding to IgG are shown as blue spheres (L112, E115, E116, W131, P132, and E133, human numbering), while the residues central for albumin binding (W51, W53, W59, and W61) are shown as red spheres. The loop containing the tryptophans is pH dependently regulated by H166 (H168 in rat) within the α2-domain (yellow spheres, human numbering). The close-ups show how H166 (H168 in rat) stabilizes the loop of amino acid residues corresponding to residues 51–61 of the α1-domain by forming charged interactions with E54 and Y60 at acidic pH. These interactions are not seen in the crystal structure solved at basic pH, which results in an unstructured loop **(B)**. The four putative N-glycosylation sites of rat FcRn are shown in orange spheres (N87, 104, 128, and 225), while only one N-glycosylation site is found in human FcRn (N102). **(D)** An amino acid sequence alignment of the cytoplasmic tail of FcRn from human, rat, and mouse. The tryptophan and the di-leucine based sorting motifs that interact with the adaptor protein 2 are highlighted in green. Amino acid residues required for calmodulin binding are marked in red (human numbering). The figures were made using PyMol, with the following PDB files; Rat FcRn pH 6.5: 3FRU (59), human FcRn pH 8.2 1EXU (60), human FcRn pH 4.2: 3MIB (61).

The neonatal Fc receptor is a transmembrane heterodimeric protein with a structure similar to that of classical and non-classical MHC class I molecules (40). Specifically, it consists of a HC with three extracellular domains (α1, α2, and α3), followed by a transmembrane part and a cytoplasmic tail. The soluble subunit β_2_m, which is common for almost all MHC class I molecules, is non-covalently paired with the FcRn HC. An overview of the crystal structures of truncated rat and human FcRn is shown in Figures 1A–C.

Inspections of solved crystal structures of soluble truncated recombinant forms of rat and human FcRn have revealed that the extracellular part of the HC has a membrane proximal α3-domain, followed by an amino-terminal α1–α2 platform that is made up of eight antiparallel β-strands with two α-helices on top (59–65). Further, β_2_m associates with the HC by making contacts with the α3-domain, located below the α1–α2 platform. Proper folding of the heterodimer in the endoplasmic reticulum (ER) is facilitated by Erp57 and calnexin, and has been shown to be a prerequisite for exit from the ER, and subsequent pH-dependent binding to the ligands (66–68).

In contrast to classical MHC class I molecules that present peptides bound in a groove located between the two α-helices on top of the α1–α2 platform, the corresponding groove is occluded in FcRn (60, 62). Instead, FcRn has evolved to bind IgG and albumin at separate binding sites on opposite sides of the α1 and α2 domains (57, 64, 69, 70) (Figure 1A). Furthermore, the human HC contains only one putative N-glycosylation site (N102) compared with four sites in the mouse and rat ectodomains (N87, N104, N128, and N225) (40, 59) (Figures 1A,B), and the differential glycosylation pattern of human and rat FcRn has been linked to different sorting and distribution in polarized cells (71). The differences in the glycosylation pattern results in molecular weights of 51 and 45 kDa for the mouse and human HC, respectively.

Crystal structures of human FcRn have been solved at both acidic and basic pH (59–61), as well as in complex with both ligands (64, 69, 72), which show that there are few structural main chain alterations in FcRn as a function of pH, except for a loop within the α1-domain that is only solved at acidic pH (60, 61) (Figures 1B,C). This loop has been shown to be important for binding of albumin (discussed below). Binding of both ligands is strictly pH dependent, with strong binding at acidic pH that becomes progressively weaker near neutral pH, suggesting that protonation of histidine residues, which is the only amino acid that changes charge between pH 5.5 and 7.4, is responsible for intra-molecular interactions or direct engagement with ligands.

The cytoplasmic tail of the HC contains conserved sorting motifs that are important for trafficking of the receptor, including di-leucine (L322/L323) and tryptophan (W311) motifs (73). An overview of the stretch of amino acids corresponding to the cytoplasmic tails of mouse, rat, and human FcRn HCs is highlighted in Figure 1D. The tryptophan motif has been demonstrated to interact with the μ subunit of adaptor protein-2 (AP-2) while the di-leucine motif interacts with the σ and γ subunits of AP-2 (74). These motifs play a role in rapid endocytosis of the receptor from the plasma membrane into endosomes, and basolateral targeting in polarized rat cells. However, as there are cross-species differences in polarized expression of FcRn in human and rodent cell lines (74–79), additional differences such as the variations in glycosylation patterns may play a role in trafficking of the receptor. Indeed, a study by Kuo and colleagues show that the difference in polarized expression between rat and human FcRn may at least partly be explained by the three additional N-glycosylation sites found in rats (71).

Another motif in the cytoplasmic tail that is conserved among species is a calmodulin binding sequence encompassing R300 and R302 (Figure 1D). Targeting of the motif by mutagenesis resulted in altered transport and decreased stability of the receptor (80). As binding of calmodulin to FcRn is highly regulated by the flux of calcium, it may be a way to modulate endosomal FcRn sorting. In addition, as calmodulin will mask a putative amphipathic α-helix in the cytoplasmic tail of FcRn, which for other proteins has been shown to be inserted into the cell membrane and induce or sense curvature, it may add another level of regulation of cellular sorting of the receptor (80, 81).

## The Structure of Albumin

**Figure 2:**
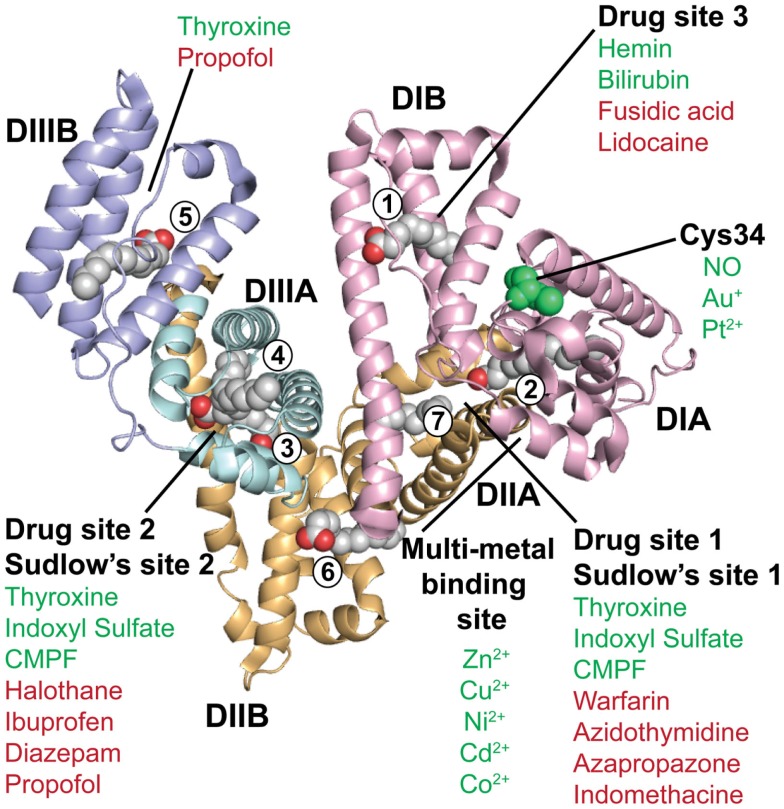
**The crystal structure of human albumin**. The illustration shows the crystal structure of human albumin solved in the presence of saturating amounts of palmitic acid. The α-helical structures of the three domains (DI, DII, and DIII) are divided into subdomains (A and B) as indicated. DI (pink) contains the fatty acid binding site 1, the free cysteine (C34), and drug binding site 3. Fatty acid site 2 is located at the interface between DI and DII. The metal binding site is located between subdomain DIA and DIIA. DII (orange) contains the drug binding site 1 (Sudlow’s site 1) as well as fatty acid sites 6 and 7. DIII (blue) contains fatty acid binding sites 3 and 4, the drug binding site 2 (Sudlow’s site 2) in DIIIA, and the fatty acid biding site 5 in DIIIB. Examples of the binding sites for endogenous and exogenous ligands for which crystal structures have been solved are listed in green and red, respectively, as reviewed in Ref. (86). The figure was designed using PyMol and the crystal structure data of human albumin solved in the presence of palmitic acid with the PDB file 1E7H (87). CMPF, carboxy-4-methyl-5-propyl-2-furanpropionic acid; NO, nitric oxide.

Albumin is exclusively synthesized and secreted into the bloodstream by liver hepatocytes to a concentration of 40 mg/ml in both mouse and man (4). It is a highly soluble and stable protein that is non-glycosylated and has a molecule weight of 66.5 kDa. X-ray crystallographic structures of human albumin has revealed that it is a heart-shaped molecule consisting of a single polypeptide of 585 amino acids with 17 pairs of disulfide bridges and 1 free cysteine (C34) (82–84). An illustration of the crystal structure of human albumin is shown in Figure 2. Albumin consists of 67% α-helices and no β-sheets, and folds into three homologous domains named DI, DII, and DIII, where each is divided into A and B subdomains (DIA, DIB, DIIA, DIIB, DIIIA, and DIIIB) (85). The domains are connected via long flexible loops.

## The FcRn–Albumin Interaction

When Chaudhury and colleagues co-eluted bovine albumin with soluble human FcRn from a human IgG-coupled column (11), this indicated that both ligands could bind the receptor simultaneously as a ternary complex. Subsequently, *in vitro* interaction analyses demonstrated this indeed to be the case. Initial mapping of the albumin binding site on human FcRn showed that a fully conserved H166, within the α2-domain of the human FcRn HC, was crucial for binding (56). An explanation for this finding was given upon scrutiny of two crystal structures of human FcRn, one of which was solved at pH 4.2, while the other was solved at pH 8.2 (60, 61). An overview of these two crystal structures are shown in Figures 1B,C. A comparison of the two structures revealed that a loop surrounding H166 within the α1-domain was only defined in the structure solved at acidic pH (61), and not in the structure solved at basic pH (60). This suggested that the configuration of the loop is pH sensitive and stabilized at acidic pH when H166 is protonated and able to form hydrogen bonds with E54 and Y60 (70) Figures 1A–C shows close-ups of the structural areas surrounding H166 and the pH sensitive loop of human and rat FcRn. The importance of these stabilizing interactions was confirmed by mutating E54 to a glutamine, which resulted in low detectable binding of albumin (70). Furthermore, the loop contains four conserved hydrophobic residues; W51, W53, W59, and W61, which are partially or fully surface-exposed (Figures 1B,C). Targeted mutagenesis of these tryptophan residues has demonstrated their fundamental role in binding of albumin, as swapping to alanine residues considerably reduced or abolished binding (72, 88). This means that the interaction is not only pH dependent but also hydrophobic in nature. In line with this is a previous report showing that the interaction has a large positive change in entropy, indicative of a hydrophobic character (57). The findings strongly support that H166 has a key regulatory role in stabilizing the loop with the cluster of tryptophan residues that directly take part in binding of albumin.

The principal binding site for FcRn on albumin was first shown to be located within the C-terminal DIII, as removal of this domain eliminated binding of albumin (70, 89). Then, targeting three fully conserved histidine residues within DIII of human albumin (H464, H510, and H535) by site-directed mutagenesis revealed that all are crucial for binding (70). In addition, mutating a lysine in position 500 (K500A), located within an extended loop that connects the two subdomains of DIII, was shown to reduce binding to FcRn by more than 30-fold (70). Furthermore, when recombinant variants of the three human albumin single domains were tested for binding to FcRn, DIII was the only domain that showed detectable binding, although with a more than 10-fold weaker affinity than full-length albumin (70). Despite limited structural knowledge, a docking model of the human FcRn–human albumin complex was built, where in addition to DIII, two exposed loops within the N-terminal DI were shown to be in proximity to the receptor (70).

**Figure 3:**
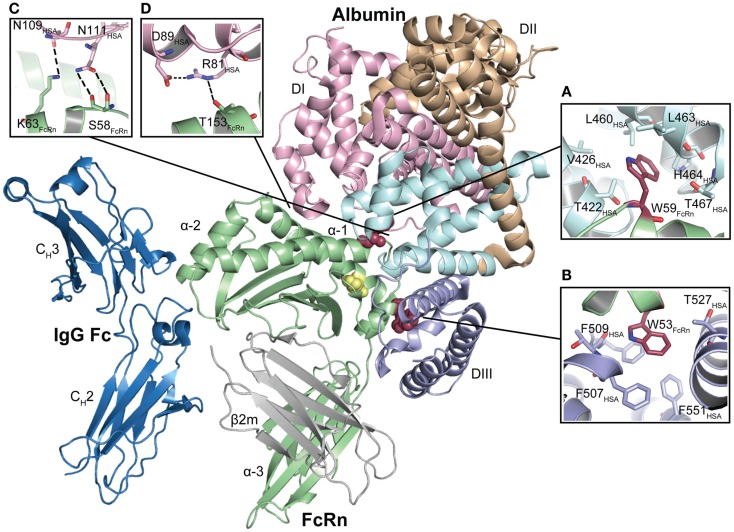
**The co-crystal structure of human FcRn bound to IgG–Fc and albumin**. The illustration shows the ternary complex of human FcRn in complex with IgG Fc and wild-type human albumin. The three domains of the FcRn HC (α1, α2, and α3) are shown in green and the β2m subunit in gray. The IgG–Fc is shown in blue while the three domains of albumin are shown in pink (DI), orange (DII), and light blue (DIII). H166 that stabilizes the structure of the loop containing W51, W53, W59, and W61 is shown in yellow. The FcRn residues W53 and W59 are shown as red spheres. **(A)** FcRn–W59 makes contacts with a hydrophobic pocket in DIIIA, which is composed of T467, T422, V426, L460, L463, and H464. **(B)** FcRn–W53 makes hydrophobic stacking with three phenylalanines in DIIIB (F507, F509, and F551). **(C)** A close-up of the structural areas of DI with N111 and N109 of albumin DI interacting with the FcRn residues S58 and K63, respectively. **(D)** A close-up of the structural interface showing the intramolecular hydrogen bond between albumin R81 and D89, and the interaction between FcRn–T153 and R81. The figures were made using PyMol and the crystal structure data of human FcRn in complex with IgG–Fc and albumin (4N0U) (69).

In agreement with these predictions, two recently published co-crystal structures of human FcRn in complex with human albumin confirmed the contributions from both DI and DIII, while DII does not take direct part in the interaction (69, 72). One of the co-crystal structures shows wild-type albumin, and the Fc part of IgG in complex with FcRn (69), and an illustration of the ternary complex is given in Figure 3. The other co-crystal shows an engineered human albumin variant (HSA13) comprising four amino acid substitutions (V418M, T420A, E505G, and V547A) (72). The latter was selected by yeast display and has considerably improved affinity for FcRn at both pH 6 and pH 7.4. The two co-crystal structures show highly similar modes of binding, but with some differences that are likely due to the introduced mutations in HSA13 DIII. However, the hydrophobic cores of the interaction interfaces are similar, and in both, the exposed FcRn–W53 and FcRn–W59 make contact with hydrophobic pockets in DIIIB and DIIIA, respectively (Figures 3A,B). More specifically, FcRn–W59 forms hydrophobic interactions with T422, V426, L460, L463, and T467 in albumin DIIIA (Figure 3A), while FcRn–W53 is inserted into a hydrophobic pocket between two α-helices in DIIIB, which comprises three phenylalanine residues F507, F509, and F551 as well as T508 and T527 (69) (Figure 3B). The crucial residue K500 interacts with E46 within the α1-domain of FcRn, as well as forming an intra-molecular interaction with E531 of DIIIB (69). Further, the interaction of FcRn–W53 with albumin requires a conformational change in albumin that comprises residues 500–510, which are part of a long loop that connects DIIIA and DIIIB. The two key residues H510 and H535 are located within this loop, and take part in the pH regulation of the FcRn–albumin interaction by stabilizing the position of the loop at acidic pH, which subsequently allows insertion of FcRn–W53 into DIIIB (69). In addition, H510 interacts with the fully conserved W176 and N173 in human FcRn (69). The third histidine, H464, is part of a hydrophobic environment that accommodates FcRn–W59 (69). Interestingly, the binding site for fatty acids within DIII overlap with the interaction sites for FcRn–W59 and FcRn–W53 (72) (discussed later).

Furthermore, both co-crystal structures show that two exposed loops within DI of albumin are in contact with residues of the α1–α2 helices of FcRn. In the wild-type complex, N109 and N111 form contacts with K63 and S58 of FcRn, respectively (Figure 3C). Albumin D89 and R81 form an intramolecular hydrogen bond, and R81 interacts with T153 of the receptor (Figure 3D). Notably, N111 has different conformations in the two co-crystals and may thus have alternative orientations. However, these studies did not experimentally address the impact of DI (69, 72). Although the docking model does not completely resemble the experimental crystal structures, it has guided the mapping of structural areas on both the receptor and albumin that are important for the interaction (70). Moreover, targeting of residues within the DI loops by site-directed mutagenesis has confirmed that the loops indeed contribute to FcRn binding (90).

Interestingly, the crystal structures also suggest that albumin may make contact with the β2m subunit, which involves long-range interactions between β2m–R12 and E50 with the side chain of albumin E505 and the backbone carbonyl of F502 (69). Again, differences are seen between the complexes containing wild-type albumin and HSA13. In one of the HSA13 complexes, K573, which is located in the middle of the last α-helix of albumin, interacts with β2m–E69 and S20 (72). Curiously, this interaction is not found in the other complexes.

Further, Oganesyan and colleagues have reported a co-crystal structure of human FcRn in complex with both wild-type albumin and an Fc fragment derived from an engineered IgG variant with improved binding to the receptor (69). As previous data have suggested, the mode of albumin binding is not altered in the presence of IgG (69).

## The Ligand Binding Properties of Albumin

The three domains of albumin contain binding pockets for a plethora of small endogenous and exogenous substances (6, 86). Thus, albumin acts as a molecule-taxi that transports essential substances and waste products in the bloodstream to their target sites. An illustration of a crystal structure of human albumin in complex with fatty acids is shown in Figure 2. Although the three domains are similar in structure, the interfaces are not, meaning that the orientation of DII relative to DI is different from the orientation of DIII relative to DII (84). In regard to binding of fatty acids, it has been shown that there are seven binding sites distributed asymmetrically throughout the tertiary structure of albumin (84, 87, 91) (Figure 2). Furthermore, crystallographic studies have shown that there are variations in the relative orientation of the domains, which likely arises from inherent flexibility of the loops and the domains. For instance, the structure of defatted human albumin has been determined in three distinct crystal forms, each of which shows different packing contacts, where the position of DIII varies (83, 84). The binding sites for different ligands and drugs are referred to either as fatty acid binding sites, drug binding sites 1 and 2, or Sudlow’s sites 1 and 2, as highlighted in Figure 2.

Under normal conditions, albumin carries 0.1–2.0 moles of fatty acids, but it can bind more in case of disease (92, 93). Structural analyses have shown that binding of fatty acids induces conformational changes that may affect binding of other ligands and drugs (94–99). As binding of FcRn engages two of the albumin domains, it raises an interesting question as to whether ligand binding to albumin will affect receptor binding and transport properties. Such knowledge is also of importance in design of novel albumin-based therapeutics, so as to control their pharmacokinetics. Interestingly, Schmidt and colleagues have shown that saturation of albumin with fatty acids interferes greatly with FcRn binding (72). This could be explained by steric hindrance, as DIII contains a high-affinity fatty acid binding site, right where the FcRn–W59 residue interacts (69, 72, 100). Thus, it is of great importance to understand whether and how different endogenous ligands and drugs are affecting the interaction with FcRn, as it may well have significant impact on transport and deposition at different body sites. The knowledge will certainly be important for design of engineered albumin variants for various applications.

In addition, albumin has one free cysteine (C34) within a pocket of DIA that is partly exposed (Figure 2). This free sulfhydryl group is a site for binding of metals (Au and Pt) and nitric oxide (101, 102), but has also been conjugated to drugs (discussed later). The C34 residue is not in proximity to the FcRn binding site on DI, but it remains to be addressed whether conjugation to this site may impact receptor binding and transport.

## FcRn as a Half-Life Regulator

The direct involvement of FcRn as a homeostatic regulator of IgG levels was first demonstrated in β_2_m-deficient mice (9, 10, 58). These mice do not express a functional FcRn and consequently have reduced levels of circulating IgG as well as rapid clearance of injected IgG (9, 10, 58). Later, inspection of mice lacking the FcRn HC revealed a similar reduction in serum levels of IgG and albumin (11, 103). These mice have serum levels of IgG and albumin fourfold to fivefold and twofold to threefold lower than that of normal mice, respectively (11, 103). Further support for the great importance of FcRn for half-life regulation was provided by a study of a rare human syndrome, named familial hypercatabolic hypoproteinemia, diagnosed in two siblings from a consanguineous marriage, who showed very low serum levels of IgG and albumin that correlated with abnormally low expression of FcRn (104). The siblings carried a mutant β_2_m subunit with an alanine to proline substitution at amino acid 11 in the signal sequence, which resulted in 80–90% reduced expression of β_2_m-associated proteins such as FcRn (12).

The plasma concentration of albumin in FcRn knock-out mice is roughly half of that in normal mice (11, 105). In humans, the average plasma concentration of albumin is 40 mg/ml (>600 μM), and as such a 70 kg person has a total albumin pool of 360 g, where 120 g constitute the intravascular albumin, which is in constant exchange with the extravascular pool. Studies in mice have demonstrated that FcRn rescues an equivalent amount of albumin in a day as the liver produces, which is estimated for adult humans to be 13 g per day (105). Thus, from an evolutionary perspective, the use of a common receptor to rescue IgG and albumin from degradation is far more economical than using energy on additional synthesis.

The serum levels of IgG and albumin are regulated by several factors including their size above the renal clearance threshold (discussed below), the balance between the rate of synthesis by plasma cells and hepatocytes, and the level of FcRn expression. Thus, if the serum level of albumin drops, the half-life should increase due to increased rescue caused by less competition for FcRn binding. This is indeed the case, and demonstrated in human studies conducted in the 1950–1970, where the half-life of radiolabeled albumin injected into people with abnormally low albumin levels, was shown to be 50–100 days (106–108). In addition, using so-called Nagase analbuminemic rats, which are genetically deficient in albumin synthesis, the half-life of injected rat albumin was measured to be 2.2 times longer than in normal rats (109, 110).

Moreover, the albumin gene exhibits a significant degree of DNA mutations causing analbuminemia or alloalbuminemia. More than 70 genetic variants have been characterized, and represent site-specific, splice-site, or frame-shift variants (111, 112). Alloalbuminemia (bisalbuminemia) is an inherited or acquired condition characterized by the presence of altered albumin variants where heterozygotes have two forms of the protein. Furthermore, analbuminemia is a rare recessive disorder in which subjects have little or no (<1 mg/ml) circulating albumin caused by a variety of mutations in the albumin gene, and is exhibited only by homozygous subjects. Although albumin is the most common serum protein, these conditions are benign, and surprisingly few biochemical effects and clinical symptoms have been observed. However, association between hypoalbuminemia and mortality has been reported for patients with diseases such as acute heart failure, renal disease, cancer, stroke, pneumonia, dementia, and hemodialysis (113–117).

Several mutant albumin variants have been reported with reduced half-life compared to wild-type albumin. One example is the Casebrook variant, which has a single point mutation in the C-terminal DIII (D494N), which introduces an N-glycosylation site. In heterozygous carriers, only 35% of total serum albumin corresponds to Casebrook (118, 119). We have recently shown that this albumin variant has a twofold reduced affinity for FcRn, which suggests that it will have shorter half-life in the presence of normal albumin that will compete more efficiently for binding to the receptor (70). This is supported by a study performed in rabbits where the D494N mutation was introduced in rabbit albumin to the effect of reducing the half-life twofold compared with normal albumin (4.7 versus 2.8 days) (119). Another example is a truncated albumin variant, named Bartin, which lacks almost the entire DIII, except for the first 25 amino acids, due to a nonsense mutation (120). This variant, when recombinantly expressed, did not show detectable binding to FcRn, which is in line with the fact that DIII is the major binding domain for FcRn (89). In fact, albumin variants with either C-terminal elongation or truncation constitute only 2–30% of the total albumin in heterozygous carriers, which has been suggested to be caused by instability of the abnormal albumin (89, 121, 122). However, it may well be due to altered or lack of FcRn binding.

## FcRn-Mediated Recycling and Transcytosis

**Figure 4:**
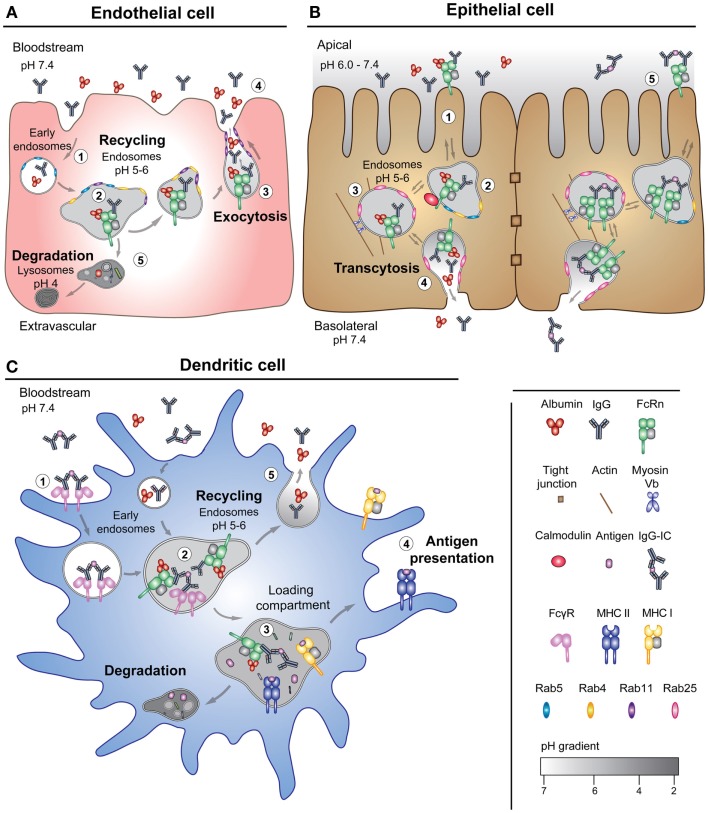
**FcRn-mediated transport pathways**. **(A)** A schematic illustration of the model of FcRn-mediated recycling pathway of its two ligands in an endothelial cell lining the vascular space. (1) IgG and albumin are taken up from the blood by pinocytosis in Rab5 positive early endosomes. (2) FcRn, predominantly localized to acidified endosomes, binds the ligands in Rab4 and Rab11 positive recycling compartments. (3) The ternary complex is recycled to the cell surface as Rab11-positive tubules, which results in exocytosis of the ligands. (4) The neutral pH of the bloodstream leads to release of the ligands. (5) Proteins that do not bind to the receptor will be sorted to late endosomes and further to lysosomes for degradation. **(B)** An illustration of a polarized epithelial cell layer and the model of FcRn-mediated bidirectional transport. (1) The acidic pH present at certain mucosal sites (apical side) may result in binding of the ligands to FcRn at the cell surface in addition to within recycling endosomes. (2) The transcytotic pathway may be regulated by calmodulin that binds to the cytoplasmic tail of FcRn, and (3) depends on the actin motor myosin Vb and Rab25. (4) Endosomes fuse with the basolateral side of the cells, which lead to release of the ligands upon exposure to neutral pH. (5) FcRn may also transcytose IgG-containing immune complexes across the polarized cell layer. **(C)** The illustration shows a DC that expresses both FcRn and classical Fcγ receptors. (1) Cross-binding of an IgG-containing immune complex to surface-expressed FcγRs leads to internalization into early endosomes. (2) The immune complexes engage FcRn within acidified endosomes. (3) FcRn directs the IgG-containing immune complexes to loading compartments for processing followed by loading of antigenic peptides onto MHC I (in terms of cross-presentation) and MHC II. (4) MHC I and II traffic to the plasma membrane for presentation of peptides to T-cells. (5) Monomeric ligands may also be recycled by DCs.

The neonatal Fc receptor transports its ligands via either of two distinct pathways, cellular recycling or transcytosis. Illustrations of the FcRn-mediated recycling and transcytotic pathways are shown in Figures 4A–C. The steps of the recycling process have been studied using advanced live cell fluorescence imaging in human endothelial cells over-expressing FcRn (123–128). These elegant studies have revealed that FcRn predominantly resides within acidified endosomes where it binds IgG that is taken up by fluid-phase pinocytosis. The low pH within the endosomes triggers binding, which results in recycling of the FcRn–IgG complex to the cell surface where exposure to an increasingly more neutral environment favors exocytosis of IgG out of the cell in a so-called kiss-and-run or prolonged-release manner (125). By contrast, proteins that do not bind FcRn will be sorted to late endosomes and subsequently to lysosomes where they are degraded (124, 126). The process is regulated by small Ras-like GTPases such as Rab4, Rab5, and Rab11 that are present on FcRn containing endosomes, as indicated in Figures 4A,B. Notably, Rab4 and Rab11 are known to be involved in recycling from sorting endosomes to the plasma membrane, whereas Rab5 is an early endosomal marker (129). Ward and colleagues have shown that during exocytosis, FcRn is sorted into tubulovesicular compartments that are positive for Rab4 and Rab11, and that only Rab11 is associated with the receptor upon exocytosis at the plasma membrane (127, 128). Importantly, the studies have revealed that there is overlap between the Rabs in the different compartments, so-called Rab conversion, as endosomes mature (130).

As FcRn binds both IgG and albumin at independent binding sites, albumin is likely to follow the same pathway as IgG, although so far, no imaging studies have been done on trafficking of the ternary complex. The non-cooperative binding of the ligands is illustrated using a high affinity 26-amino acid peptide dimer SYN1436. The peptide binds to the IgG site of the receptor and blocks FcRn-mediated transport of IgG, resulting in an 80% reduction in serum levels of IgG in cynomolgus monkeys without reducing serum albumin levels (131, 132).

Furthermore, this raises the question of which cells and organs that contribute to FcRn-mediated salvage. So far, it has been shown that both non-hematopoietic and hematopoietic cells are of equal importance (49, 51). Studies in mice have identified endothelial cells and myeloid-derived professional antigen presenting cells as key players (49). When the expression of FcRn was conditionally deleted from these cell types, the serum levels of IgG and albumin were reduced by fourfold and twofold, respectively (49, 51). The contribution of the different cell types will depend on the rate of uptake, the level of FcRn expression, and the abundance of the ligands. In addition, the expression of the receptor can be up- or down-regulated by pro-inflammatory substances and cytokines, which add another level of regulation (133, 134).

As initially described, FcRn binds IgG derived from the mother’s milk and mediates transcytosis across the neonatal rat intestine (135–137). While the expression of FcRn in the rodent intestine is developmentally down-regulated, it is constitutive in humans throughout life (21, 138, 139). Several studies have shown that human FcRn can transport both monomeric IgG and IgG-containing immune complexes across mucosal epithelial barriers in both directions, a process that also relies on a pH gradient (19, 20, 23, 140, 141).

Using polarized Madin–Darby canine kidney cells that over-express human FcRn, it has been demonstrated that the receptor transports IgG from either apical or basolateral side into the recycling endosome (75, 78). As shown in Figure 4B, the actin motor myosin Vb and the GTPase Rab25 regulate a sorting step that determines transcytosis without affecting recycling (142). In addition, it was demonstrated that Rab11 is dispensable for transcytosis, but regulates recycling to the basolateral side (142).

These findings raise the question of whether or not FcRn is capable of mediating transcytosis of albumin across the same cellular barriers, and whether the stoichiometry of the interaction with FcRn plays a role, as albumin binds FcRn in a 1:1 manner, while IgG is homodimeric and has two binding sites for FcRn (11, 60). This is interesting, as albumin transport across epithelia would allow for delivery of albumin-based therapeutics. However, no *in vitro* cellular studies have so far demonstrated that FcRn can transport albumin efficiently alone or in the presence of IgG across polarized cells. One study using MDCK cells over-expressing rat FcRn could not detect transcytosis of rat albumin (78). Several studies have demonstrated that IgG-based fusions, vaccines, and nanoparticles can be delivered in an FcRn-dependent manner across mucosal barriers (25–32). Interestingly, in mice, albumin was found in saliva (1–3.0 μg/ml), feces (0.1 mg/ml), and the small intestine (0.5 mg/ml) (143), and in a human study, more albumin (655.0 μg/ml) than IgG (50.0 μg/ml) was found in the fluid of the respiratory tract (144). Illustrations showing the models for FcRn-mediated recycling and transcytosis are given in Figures 4A–C.

## The Role of FcRn in the Kidneys

**Figure 5:**
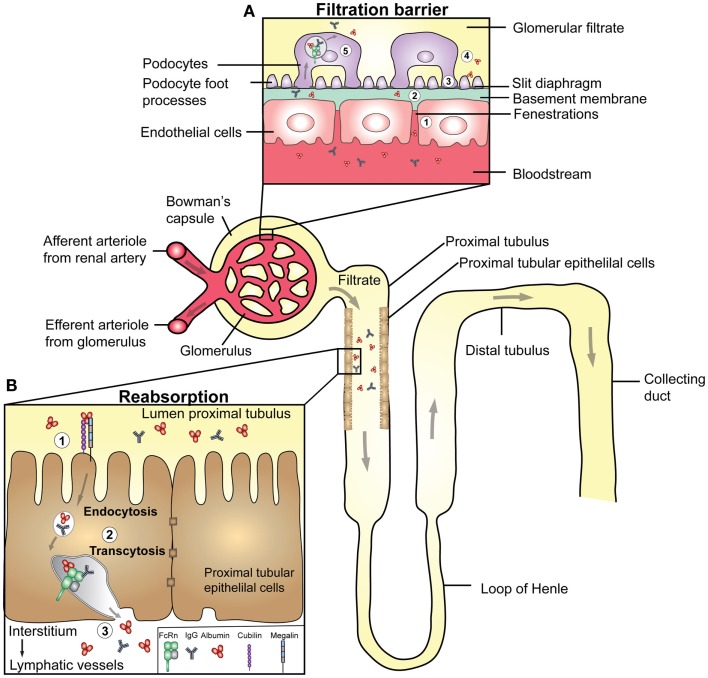
**Transport of IgG and albumin in the kidneys**. A schematic cartoon showing the nephron of the kidney and transport pathways. Blood enters the kidneys through the afferent arteriole from the renal artery, proceeds through the capillaries of the glomerulus where filtration occurs, and exits through the efferent arteriole. **(A)** The filtration barrier of the glomerulus. (1) The first barrier is the fenestrations between endothelial cells of the glomerular capillaries. (2) The second barrier is the basement membrane, a non-cellular layer consisting of extracellular matrix molecules, which make up charged pores. The podocytes are specialized epithelial cells that encapsulate the capillaries and the basement membrane, and form the outermost layer of epithelial cells facing the glomerular filtrate. (3) The foot processes of the podocytes have slits between them (slit diaphragms) that form the third layer of the filtration barrier. (4) As the pore size of the barrier is between 60–70 kDa, close to the size of albumin (66.5 kDa), some albumin passes the filter. (5) Podocytes express FcRn, and may transcytose IgG and albumin to the glomerular filtrate to prevent clogging of the filter. **(B)** The glomerular filtrate enters the proximal tubuli where proximal tubular epithelial cells lining the lumen of the tubuli are involved in reabsorption of albumin and IgG, and thus prevent loss into the urine. (1) Proximal tubular epithelial cells also express the cubilin/megalin receptor complex that binds albumin. (2) In acidified endosomes, FcRn binds the ligands, and facilitate transcytosis. (3) The ligands are delivered to the interstitial space of the kidneys followed by drainage to lymphatic vessels and re-entry to the blood circulation.

Serum persistence of soluble proteins also depends on the renal clearance threshold, which prevents clearance of proteins larger than 60–70 kDa from the body through the urine. The nephron, its filtration barrier, and a model for protein reabsorption are illustrated in Figure 5. Specifically, the kidneys receive blood from the renal arteries, which reach the glomerular filtration barriers that form three size- and charge-selective filters. These block the passage of cells and larger proteins into the urine (145). First, the blood is filtered in the glomerular capsule of the nephrons, and then the filtrate is transported via the proximal convoluted tubule where water and essential proteins are reabsorbed. As the kidneys filter roughly 180 l of blood each day, it means that astonishing >7 kg of albumin and >2 kg of IgG are processed by the kidneys.

The first study addressing a potential role of FcRn in the kidneys showed that the receptor is expressed in specialized epithelial cells named podocytes (46), which are part of the glomerular capsule, and the filtration barrier (Figure 5A). Akilesh and colleagues demonstrated that IgG accumulated at this barrier in mice lacking FcRn (50). Based on this finding, FcRn-positive podocytes were postulated to be the key cells responsible for removal of IgG from the glomeruli. Notably, delayed clearance of IgG may lead to serum-induced nephritis, which may indicate that the receptor could be involved in the pathology of diseases such as systemic lupus erythematosus, where kidney damage is commonly caused by deposition of IgG and immune complexes at the glomerular barrier. Thus, the presence of FcRn may provide a mechanism to clear IgG from the glomerular basement membrane and deliver it intact into the glomerular filtrate. Although the same study did not address how albumin is handled, it has been shown that podocyte-like epithelial cells endocytose albumin that co-localizes with FcRn (146). The glomerular filtrate that enters the proximal convoluted tubule is well known to contain albumin, and it has been demonstrated that albumin is retrieved by proximal tubule cells (147). These cells also express FcRn (46), and several lines of evidence support an important role of FcRn in retrieval of albumin (148–150). The transport pathways of IgG and albumin in the nephron are shown in Figure 5.

It has been demonstrated that FcRn-deficient mice secrete more albumin into the urine than normal mice, and the same was observed for normal mice transplanted with a kidney lacking FcRn (150). When FcRn-deficient mice received an FcRn-expressing kidney by transplantation, increased serum levels of albumin were observed. In contrast, FcRn-deficient mice showed minimal urinary excretion of IgG, which increased to normal levels when knock-out mice were transplanted with a single FcRn expressing kidney (150). This may imply that the glomeruli get clogged with IgG in the absence of FcRn, while it is otherwise transported by the podocytes.

Furthermore, recent studies using a transgenic mouse strain with podocyte-specific expression of doxycycline-inducible tagged mouse albumin showed that albumin secreted into the filtrate is subsequently reabsorbed by proximal tubular cells, which resulted in increased serum levels (149). In addition, deletion of FcRn expression abolished uptake of albumin, and also IgG in proximal tubular cells (148, 149). Collectively, these data support a key role for FcRn in the kidney tubular system, where FcRn protects albumin and IgG from being excreted into the urine, a mechanism that likely relies on directed pH-dependent transcytosis of intact ligands back to the circulatory system. Thus, the data point to the first evidence of FcRn-mediated transcytosis of albumin as part of a ternary complex with IgG.

However, the mechanisms responsible for handling albumin in the kidneys may not necessarily rely on FcRn only, as several studies claim that uptake of albumin by the proximal tubular cells is an active process that depends on a receptor complex consisting of cubilin and megalin (151–154) (Figure 5B). Homozygous deletion of cubilin in mice is lethal, and studies done in heterozygous individuals showed reduced renal proximal tubular uptake, increased urinary loss, and decreased blood levels of albumin (152, 153). Further, dogs with a defective cubilin gene excrete large amounts of albumin due to an almost complete lack of reabsorption (153). In addition, cubilin has been shown to associate with megalin, and in line with this, both mice and humans deficient in megalin expression have reduced tubular reabsorption of albumin (153, 155, 156). As illustrated in Figure 5B, these findings thus raise the possibility that FcRn may work in concert with the megalin and cubilin complex. Such a pathway may then serve as a selective process where only albumin with intact receptor binding properties are returned to the blood, while albumin with bound ligands is excreted in the urine or sorted to intracellular compartments destined for degradation.

## A Regulatory Role of FcRn in the Liver?

The liver receives six times more blood per day than the kidneys due to supply of both arterial blood from the heart, and venous blood from the digestive tract. The main cell type (70–80%) of the liver is specialized epithelial cells named hepatocytes, which have an apical side facing the bile canaculi, and a basolateral side that is in contact with the blood (157). This means that large amounts of IgG and albumin are in direct contact with endocytically active hepatocytes. As such, it is of great interest to address the importance of IgG and albumin receptors including FcRn in liver cells in the context of IgG and albumin homeostasis.

Despite the fact that the amount of IgG in blood is five times higher than that of IgA, astonishingly little IgG enters the bile compared with secretory IgA (158). Likewise, even though albumin is produced by hepatocytes that face both blood and bile ducts, the level of albumin in blood is 100-fold higher than that found in the bile (158, 159). However, there is a scarcity of data on which transport mechanisms operate in hepatocytes to maintain serum to bile ratio of IgG and albumin, rescuing these ligands from catabolism and directing newly synthesized albumin to the blood. Only a few papers have addressed the expression and function of FcRn in the liver (53, 160, 161). One shows expression of FcRn in the rat liver and that it binds IgG in a pH-dependent manner (53). Thus, there is a great need for studies addressing the impact of FcRn in the liver, so as to understand the biology and biodistribution of IgG and albumin. In this context, it is highly interesting that the liver is the principal site for albumin synthesis. Studies addressing these aspects will shed new light on how to avoid liver toxicity.

## The Role of FcRn in Immune Cells

The neonatal Fc receptor is expressed in mouse and human myeloid-derived antigen presenting cells such as monocytes, macrophages, and dendritic cells (DCs) (42, 49, 51). Besides being of importance in half-life regulation, FcRn in these cells has a role in antigen presentation following uptake of IgG-containing immune complexes (162, 163). The different sorting pathways for IgG and IgG-containing immune complexes are illustrated in Figure 4C. Whereas monomeric IgG is efficiently recycled by FcRn in DCs, IgG-immune complexes are taken up following binding to classical Fcγ receptors expressed on the cell surface, before FcRn is recruited within acidified endosomes, followed by transport to a degradative pathway leading to antigen loading on either MHC class I or II for presentation to T cells (162, 163) (Figure 4C). In particular, FcRn has been shown to be of great importance in cross-presentation mediated by monocyte-derived CD8−CD11b+ DCs, a pathway that was recently shown to be critical in cancer protection in mice (164). The role of FcRn in antigen presentation has been thoroughly reviewed elsewhere (163, 165).

Notably, the invariant chain (Ii) is an important regulator of MHC class I and II transport (166, 167), and one study showed FcRn association with Ii in DCs as well as in cytokine-stimulated epithelial and endothelial cells (168). The association of the FcRn–Ii complex was initiated within the ER, and Ii association targeted the receptor to late endosomes or lysosomes. Thus, Ii may be a modulator of FcRn trafficking of immune-complexes (168).

As efficient uptake and delivery of immune complexes to FcRn is dependent on initial binding to the Fcγ receptor, it is of interest to address whether albumin fusions or complexes are excluded from this pathway or not. Interestingly, albumin has been shown to be efficiently taken up by phagocytes of sentinel lymph nodes in mice, and when conjugated as a vaccine it resulted in increased accumulation in the lymph node followed by enhanced T-cell priming and anti-tumor efficacy (169).

## The Role of FcRn at Immune-Privileged Sites

Immune-privileged sites are characterized by limited or absence of immune surveillance and include the central nervous system, the eyes, testis, and placenta. With regard to FcRn, confocal microscopy studies have identified its expression in choroid plexus epithelium, which forms part of the blood–brain-barrier (BBB) (170), a finding, which suggested that the receptor may mediate efflux of IgG from the brain to the blood in a process of reverse transcytosis (171, 172). Indeed, in a murine model of Alzheimer’s disease, it was shown that FcRn expressed within the BBB is involved in the removal of amyloid β-peptide specific IgG-immune complexes from the brain of older mice (173). However, another study is in conflict with this, and claims that FcRn is not responsible for the low levels of IgG in the brain relative to that found in plasma (174, 175). A more recent study, where IgG variants were injected intracranically in rats, showed that an IgG with improved FcRn affinity was removed faster from the brain compared to a mutant with no affinity for the receptor, which was present for a longer time post injection (172). Therefore, further studies are required to elucidate the function of FcRn at the BBB.

The neonatal Fc receptor is also found to be expressed in the eyes in a variety of tissues such as the cornea, lens epithelium, and retinal blood vessel, but not in the retinal pigment epithelium and the choroid (44, 52). Investigation in normal mice and mice deficient for FcRn has demonstrated that the receptor plays a role in removing intravitreally administered IgG via the blood retinal barrier for delivery into the blood in analogy to the process of reverse transcytosis described for the BBB (176). Another recent study shows that retinal endothelial cells express FcRn, which may imply that it is preventing IgG from transport across the blood–retinal barrier (44).

As albumin is present in blood in higher levels than IgG, the immune-privileged sites are exposed to both ligands; however, none of the above studies addressed the importance of FcRn on albumin transport at these barriers. In addition, the importance of FcRn in the testis has yet not been addressed.

## FcRn: A Regulator of Transplacental Transport

The transfer of passive immunity from mother to offspring was first attributed to the transfer of IgG across the intestine in neonatal rats via FcRn-mediated transcytosis, as discussed above. In humans, IgG is delivered via the placenta, and a human ortholog of FcRn was first cloned from specialized placental cells named syncytiotrophoblasts, and later these cells were shown to bind IgG (22, 24, 177). However, to enter the fetal blood, IgG needs to cross not only the syncytiotrophoblasts but also the fetal endothelium. In addition to FcRn, another IgG binding receptor, Fcγ receptor IIB, has been identified in placental endothelial cells and postulated to be involved in the shuttle of IgG to the fetus, but conflicting data exist (178–181). Using *ex vivo* placenta perfusions, it was demonstrated that an IgG molecule with no affinity for classical Fcγ receptors is transported across the placenta, while an IgG with no affinity for FcRn is not (24). In addition, an engineered IgG variant with improved affinity for FcRn was transported more efficiently than wild-type IgG (182). These data strongly suggest that transport of IgG is solely dependent on FcRn.

With regard to transfer of albumin, the literature is conflicting. Early studies from the 1950–1960s conclude that transport of albumin to the fetus does not occur in mice, rats, and dogs (183–185), while reports from studies in rabbits and rhesus monkeys show that albumin is transported, although to a much lesser extent than IgG (183, 186–188). More specifically, the levels of albumin in the fetus of rabbits were found to be 40% of that of the levels of maternally derived IgG (187), while in rhesus monkeys 15- to 20-fold lower levels of albumin were detected compared with IgG (186). In a human study from 1964, where radioiodinated IgG and albumin were injected into pregnant women in the last month of gestation, the levels of albumin in the offspring were measured to be only 14–15% of that found in the maternal blood, whereas the amounts of IgG were slightly higher than that found in the mothers (189). These studies indicate that there are differences across species, and that albumin is inefficiently transported across the placenta compared with IgG in rhesus monkeys and humans. Whether or not other albumin binding receptors are involved in albumin transport in the different cellular layers of the placenta is not known, but two studies have identified the cubilin/megalin complex in human syncytiotrophoblasts (190, 191). Hence, there is a great need for in-depth studies addressing how albumin and albumin-bound cargo are handled by the cells of the placenta. For instance, the use of *in vitro* perfusion systems of human placenta combined with current molecular methods may facilitate new insights into the mechanisms at play.

## The Impact of Cross-Species Binding Differences

As rodents are routinely used for pre-clinical screening of IgG and albumin-based therapeutics prior to studies in primates, it is necessary to understand how human IgG and human albumin bind mouse and rat FcRn. Indeed, large differences in cross-species binding exist that need to be considered prior to *in vivo* evaluation of their pharmacokinetic and pharmacodynamic profiles (17, 18, 192). The importance of such cross-species differences was first appreciated when it was found that human FcRn does not bind to mouse IgG, while mouse FcRn binds human IgG more strongly than mouse IgG, a finding that explains why human IgG has a longer serum half-life in wild-type mice than mouse IgG (192–194). On the other hand, the lack of binding of mouse IgG to human FcRn gives an explanation for the disappointingly short *in vivo* persistence of therapeutic mouse IgG in humans (195, 196). For *in vivo* evaluation of human IgG therapeutics, the state-of-the-art mouse strains used are genetically modified as they lack expression of mouse FcRn and instead are transgenic for human FcRn (193, 197, 198). Injected human IgGs have a long half-life in such mice, but their serum levels of endogenous IgG are low due to lack of binding to mouse IgGs. A more complete and attractive model would be mice that are transgenic for human IgG while also express human FcRn.

Regarding albumin, recent studies have demonstrated that FcRn from both mice and humans bind more strongly to mouse albumin than to the human form, and that mouse FcRn binds very poorly to human albumin (17, 18, 199). These findings are in agreement with the fact that mice transgenic for human FcRn show higher levels of mouse albumin in blood than normal mice (11), and that the serum half-life of human albumin is similar to that of a human albumin variant (K500A) with considerably reduced affinity toward human FcRn (18). Another example is that the half-life of human albumin is only 15 h in normal rats compared to 49 h for rat albumin (200). Consequently, rodents have limitations as pre-clinical models for evaluation of human albumin variants and albumin-based therapeutics, as injected variants will be ignored by mouse FcRn in the presence of 40 mg/ml of endogenous mouse albumin. Similarly, human FcRn transgenic mice will rescue mouse albumin from degradation more efficiently than injected human albumin. These are critical matters to consider prior to evaluation of human albumin-based therapeutics. Thus, there is a great need for construction of novel mouse strains that are human FcRn transgenic and lack expression of mouse albumin, or where the gene for mouse albumin is replaced with the human counterpart.

## FcRn and Therapeutic Applications

The versatile roles of FcRn are increasingly appreciated by both academic labs and biotech companies, as new opportunities for therapy become available. Many examples of utilization of the biology of FcRn have given rise to new classes of IgG-based therapeutics. In light of the expanding interest and mapping of the relationship between FcRn and albumin, it is likely that such knowledge will pave the way for tailoring of albumin-based therapeutics.

As IgG is the fastest growing class of biotherapeutics, there is a great interest for optimization of effector functions and *in vivo* efficacy by manipulating the interaction with FcRn. As the interaction is pH dependent, the major challenge has been to improve binding without disrupting pH-dependent binding. Despite this, several examples exist on successful engineering by targeting amino acid residues at the core or near the FcRn interaction site on IgG (201–206).

The same principle has just been revealed for human albumin, by showing that swapping of part of DIII or introduction of substitutions within DIII result in albumin variants with altered FcRn-binding properties (18, 70, 72, 89, 199). The first example of engineering was demonstrated by swapping DIII from mouse albumin onto DI–DII of human albumin, which resulted in a hybrid albumin with considerably improved binding toward mouse and human FcRn, whereas swapping of DIII of human albumin onto DI–DII of mouse albumin reduced binding considerably (199). Moreover, swapping of a stretch of amino acids corresponding to the last C-terminal α-helix of mouse albumin onto the human counterpart gave rise to fourfold improved binding (199).

In another approach, yeast display was used to develop human albumin variants with a range of affinities toward human FcRn. One such variant (E505G/V547A) gained more than 10-fold improved affinity at pH 6.0 with a minor increase at neutral pH, which extended the half-life in human FcRn transgenic mice and cynomolgus monkeys by 1.5-fold and 1.3-fold, respectively (72). Notably, selected variants with three and four DIII substitutions were immunogenic when injected into human FcRn transgenic mice (72).

Furthermore, using an approach based on structural analysis and cross-species binding analyses, a single substituted human albumin variant (K573P) was identified with 12-fold improved affinity toward human FcRn at acidic pH without detectable binding at neutral pH (18). When evaluated in mice transgenic for human FcRn and cynomolgus monkeys, the engineered variant showed 1.4 and 1.6-fold extended half-life, respectively. Interestingly, replacement of K573 with any amino acid resulted in enhanced binding to human FcRn at acidic pH, a finding that is not easily explained based on the available co-crystal structures (18, 69, 72). Also, from an evolutionary perspective, it is interesting that all species have a proline at position 573, except for humans and orangutans. Notably, introduction of K573P in human albumin improved binding to mouse FcRn by more than 20-fold, demonstrating the importance of a proline at this position for optimal binding to mouse FcRn, and it explains partly why human albumin binds the mouse receptor poorly (17, 18). Interestingly, mutating amino acid residues in a loop of DI to alanines resulted in HSA variants with slightly improved binding to the receptor, and combining mutations in DI and DIII may thus give rise to variants with further improved FcRn binding properties (90).

## Albumin Targeting and Therapeutics

The therapeutic efficacy of small proteins, peptides, and chemical drugs is hampered by short *in vivo* serum half-life as they are cleared rapidly by the kidneys or the liver. Two strategies for rescue are fusion to Fc or albumin. Such approaches have been extensively explored, and Fc-fused drugs are approved for clinical use. An example is Fc-fusion to the tumor necrosis factor (TNF) receptor (Etanercept, Enbrel^®^), which blocks binding of TNF-α to cellular TNF receptor and thus inhibits pro-inflammatory activity in rheumatoid arthritis patients (207). Fc-fused therapeutics and vaccines have also been shown to cross mucosal barriers in an FcRn-dependent manner (25–32). Although such fusions bind FcRn, the biophysical nature of the fused drug may alter binding to FcRn (208).

In regard to albumin, its properties have been utilized in five distinguished drug delivery technologies; (1) genetic fusion to the N- or C-terminal end, (2) chemical coupling of low-molecular weight drugs, (3) association of drugs with hydrophobic pockets of albumin, (4) association of albumin-binding domains (ABDs) that are genetically fused to drugs, and (5) encapsulation of drugs into albumin nanoparticles (15, 16).

A number of reports exist on genetic fusion of therapeutic proteins to wild-type albumin. Examples are hirudin (209), CD4 (210), insulin (211), growth hormone (212), granulocyte colony stimulating factor (213), α and β interferons (214–216), and antibody fragments (217–221). All have shown improved pharmacokinetics compared to non-fused counterparts. For instance, recombinant interferon α2a has only a half-life of 4 h in humans, which is increased to 141 h when fused to albumin (214). Another example is recombinant coagulation factors that have very short half-life in humans. For instance, commercially available recombinant FVIIa (NovoSeven^®^) has a half-life of only 2.4 h (222). As a result, patients require multiple and frequent infusions to manage bleeding episodes. But when the factors are fused to the N-terminal end of albumin via flexible glycine serine linkers, they maintain activity and gain considerably extended half-life in both pre-clinical animals and humans (222–224). Such fusions have now entered clinical trials for treatment of hemophilia (225). In addition, the first albumin fusion that has entered the market is a fusion of glucagon-like peptide-1 (GLP-1) to wild-type albumin (Eperzan^®^/Tanzeum^®^), which was approved for treatment of type II diabetes in 2014 (226, 227). These examples pinpoint that the albumin fusion platform is a successful strategy for improving the *in vivo* efficacy of small therapeutic proteins. In addition, a favorable feature of genetic fusion is that it allows a simple one-step synthesis process with no need for *in vitro* chemical cross linking steps.

Such fusions were designed and constructed long before the relationship between albumin and FcRn was appreciated. This raises the question of whether genetic fusion of proteins to the N or C-terminal end of albumin, or both, compromises pH-dependent binding to FcRn. One study has so far addressed this concern, where direct fusion of a peptide or an antibody single-chain variable fragment (scFv) via a linker to the N-terminal end gave no or only a minor reduction in binding affinity at acidic pH, whereas fusion to the C-terminal had a more pronounced effect and at most twofold weaker affinity (199). Although the decrease in binding was minor, it may play a role *in vivo* when fusions are injected into animals or humans where it will compete for binding to FcRn in the presence of 40 mg/ml of endogenous albumin. Thus, binding to FcRn should be addressed for each fusion as the nature of various fusion partners may affect receptor binding differently.

The presence of high amounts of albumin at the site of tumors and inflamed tissues has been utilized for tumor targeting by chemical conjugation of drugs to albumin (228). One example is methotrexate for treatment of renal carcinomas and autoimmune diseases such as rheumatoid arthritis (229–231). It is likely that random conjugation of payloads to surface exposed amino acid residues on albumin will negatively affect clearance and FcRn transport. Another approach is nab-paclitaxel (Abraxane^®^), which is composed of the lipophilic drug paclitaxel that is encapsulated with albumin under high pressure. The drug was approved in 2005 for treatment of metastatic breast cancer, and is currently in trials for treatment of non-small lung cancer, pancreas cancer, and melanoma (16, 232–236). Following administration, the nanoparticles dissociate and paclitaxel becomes associated with albumin in blood. Whether nanoparticles containing albumin are capable of interacting with FcRn remains to be addressed.

A more specific way to chemically target albumin is to utilize the free C34 on the DI of albumin. For instance, in the technology known as Drug Affinity Complex (DAC^®^), drugs are specifically and stably conjugated to either exogenous or endogenous albumin (237). One such DAC-based drug is exendin-4, which is a GLP-1 homolog (CJC–1131) for treatment of type 2 diabetes that has entered clinical trials (238–240). The power of this technology is mirrored by the pharmacokinetics in humans, where the half-lives of GLP-1 analogs have been shown to be a few hours compared with 9–15 days for the C34-bound drug (241). Another example is an acidic sensitive prodrug of doxorubicin (Aldoxorubicin) that is rapidly bound to C34 after intravenous administration (237). The drug is conjugated via a linker that is cleaved upon exposure to an acidic environment as found in tumor tissues, and Aldoxorubicin is currently in clinical trials for treatment of sarcoma and glioblastoma (16). Whether the acidic-sensitive linker is protected or cleaved during FcRn-mediating transport remains to be investigated.

A more recent example is a designed ankyrin repeat protein (DARPin) with specificity for the epithelial cell adhesion molecule that has been modified in the N-terminal end by introduction of the non-natural amino acid azidohomoalanine. The modification enables linkage of site-specifically dibenzocyclooctyne to C34 of wild-type mouse albumin (242). The conjugate was shown to bind mouse FcRn, a strategy that extended the serum half-life of the DARPin from 11 min to 17.4 h in mice (242). As discussed above, targeting of C34 will presumably not interfere with FcRn binding and transport.

Albumin may also be utilized by reversible non-covalent association. Such a strategy excludes the need for *in vitro* conjugation as endogenous albumin is targeted post injection (243–245). One strategy is to utilize fatty acids as tags that can be conjugated to drugs, and association with albumin post injection, which results in extended half-life. An example is conjugation of a myristate tag to a lysine residue on the insulin analog detemir (Levemir^®^), which is approved for treatment of diabetes types 1 and 2 (243, 246, 247). At the time of subcutaneous administration, the drug exists as hexamers that dissociate into monomers in blood and associate with circulating albumin. The procedure prolongs the half-life from 5–6 min for the peptide to 5–7 h for the albumin-targeted drug (15). Similar strategies using fatty acids as tags with improvement in pharmacokinetics have been described for insulin-based drugs (Tresiba^®^ and Victoza^®^) (248, 249).

Furthermore, an albumin-binding minimal organic molecule [2-(3-maleimidopropanamido)-6-(4-(4-iodophenyl)butanamido) hexanoate] that has been chemically conjugated to a free cysteine residue engineered into the C-terminal end of an scFv fragment with tumor specificity, was shown to increase the half-life from 0.5 to 16.6 h in mice (250). In addition, the modified scFv showed superior tumor accumulation in tumor-bearing mice (250).

An alternative is to target albumin with anti-albumin binding antibody fragments (221). An illustrating example is a bi-specific F(ab)2 fragment with one-arm that targets TNF and one that targets albumin, which showed a half-life fivefold longer than that of mono-specific anti-TNF F(ab)2. This half-life is comparable to that of rat albumin itself (42.5 versus 49.1 h, respectively), which supports that the anti-albumin Fab does not interfere with FcRn binding and transport (221).

The same has been demonstrated using small albumin-binding domain (ABD) antibodies (11–13 kDa; AlbudAb^®^) selected to bind rat albumin with high affinity (13 nM). The half-life in rats is 53 h, which is equal to the half-life measured for albumin (53 h) (200). Notably, it has been demonstrated that this AlbudAb binds to the DII of albumin, which is not engaged in FcRn binding (200, 251). So-called nanobodies with specificity for albumin have also been selected (252).

Moreover, small peptides with specificity for albumin from several species have been selected using phage display technology, where the core sequence (DICLPRWGCLW) is functionally dependent on a disulfide bridge between the two cysteine residues (253). One of the selected peptides (SA21) had a half-life of 2.3 h in rabbits, significantly longer than the 7.3 min of an unrelated peptide of similar size (253). Moreover, when albumin binding peptides with a wide range of affinities were fused to a Fab, with specificity for human epidermal growth factor receptor 2 (HER2) (AB.Fab4D5) derived from the clinically approved trastuzumab (Herceptin^®^), a correlation between albumin affinity and serum half-life was demonstrated, as fusions with peptides of low affinity were eliminated faster than fusions with strongly binding peptides (254). These data show that the pharmacokinetics of a protein of interest may be tailored as a function of albumin affinity.

Furthermore, AB.Fab4D5 has been tested for its ability to target HER2-positive tumors in allograft mouse models, and demonstrated rapid tumor targeting in addition to elimination from the blood faster than the parental antibody (255). Thus, a significantly improved tumor to normal tissue ratio was achieved. Notably, Fab4D5 accumulated in the kidneys, while AB.Fab4D5 did not, suggesting that the albumin binding peptide has a great impact on biodistribution and organ deposition (255). These effects may be explained by the increase in size above the threshold for renal clearance and maintained albumin binding to FcRn.

Another approach has been to utilize the ABD derived from *Streptococcus* protein G. For instance, anti-HER2 Fab4D5 has been genetically fused via its light chain C-terminal end to such an ABD, which prolonged the half-life to 21 h compared to only 2 h for the naked Fab4D5 in mice, and comparable to that obtained with Fab4D5 fused to albumin targeting peptides (255, 256). Also, less ABD-fused Fab4D5 accumulated in the kidneys relative to naked Fab4D5.

A similar approach has been used to improve the pharmacokinetics of a divalent anti-HER2 Affibody (Z_HER2:342_) molecule genetically fused to ABD. An Affibody is a small scaffold (~7 kDa) derived from the IgG binding domain of *staphylococcal* protein A that is used for construction of combinatorial libraries and target selections (257). Interestingly, high tumor uptake of radiolabeled anti-HER2 Affibody (Z_HER2:342_) fused to ABD was demonstrated in HER2-positive microxenograft mice, where 25-fold reduction in kidney accumulation compared with the Affibody lacking ABD was observed (258). Thus again, non-covalent association with albumin was used to redistribute the therapeutic agent to avoid kidney accumulation. In regard to FcRn, it has been demonstrated that ABD, both alone and when fused to anti-HER2 Affibody (Z_HER2:342_), binds albumin independently of pH-dependent binding to FcRn (259). Furthermore, FcRn binding was unaffected by the presence of IgG, and the ABD fusion showed a similar biodistribution profile as rat albumin in wild-type rats (259). Thus, these data strongly indicate that the anti-albumin peptides and ABD bind FcRn properly when fused to a protein of interest. However, this should be addressed in each case as to rule out that the fusion partner causes steric hindrance or otherwise negatively affect FcRn binding. Importantly, to allow efficient FcRn-mediated recycling and transcytosis, the albumin binding molecules need to bind albumin not only at physiological pH but also at the mildly acidic pH found within endosomal compartments. The latter was addressed for the albumin binding peptides, where no differences in affinity were detected as a function of pH (253).

## Concluding Remarks

The neonatal Fc receptor is a unique cellular receptor with affinity for IgG and albumin, two completely unrelated plasma proteins. Both ligands bind the receptor in a remarkably similar pH-dependent manner, which is fundamental for the versatile functions spanning both immunological and non-immunological processes. It is broadly expressed and functions in both hematopoietic and non-hematopoietic cells, including specialized cell types of vital organs such as the kidneys, liver, and placenta, which highlight the importance of the receptor in homeostatic regulation of the ligands throughout the body. However, further studies are needed to obtain a more complete understanding of the roles of FcRn at every body site, and also the potential impact of other albumin receptors.

Furthermore, our molecular understanding of how FcRn binds its ligands and controls their half-life has prompted engineering of new classes of IgG molecules with unique blocking and transport properties. The recent elucidation of the FcRn–albumin relationship also offers opportunities for refining existing albumin technologies or development of completely new concepts. Recently, engineered albumin variants with altered FcRn binding kinetics were shown to result in extended serum half-life beyond that of natural albumin. This has triggered enthusiasm as these may be used as fusion partners to gain superior pharmacokinetics of both biotherapeutics and chemical drugs. These findings will surely encourage further molecular engineering of albumin variants with tailored pharmacokinetic properties. Recent knowledge about the FcRn–albumin interaction and its key role in homeostatic regulation will also allow reinterpretation of previously published data on conjugation of payloads, as disruption of the interaction with FcRn will undoubtedly have a major impact on their *in vivo* behavior. Especially, pre-clinical *in vivo* studies of human albumin-based therapeutics in mice or rats need to be reassessed since rodent FcRn binds only weakly to human albumin, which will compete poorly for binding to the mouse receptor in the presence of larger amounts of endogenous mouse albumin. Thus, for future studies, there is a great need for new transgenic pre-clinical animal models suitable for studies of human albumin variants and human albumin-based therapeutics.

## Conflict of Interest Statement

The authors declare that the research was conducted in the absence of any commercial or financial relationships that could be construed as a potential conflict of interest.

## Abbreviations

β_2_m, β_2_-microglobulin; AP-2, adaptor protein 2; ABD, albumin-binding domain; BBB, blood–brain-barrier; DCs, dendritic cells; DARPin, designed ankyrin repeat protein; ER, endoplasmic reticulum; FcRn, neonatal Fc receptor; GLP-1, glucagon-like peptide-1; gp, glycoprotein; HC, heavy chain; HER2, human epidermal growth factor receptor 2; IgG, immunoglobulin G; Ii, invariant chain; MHC, major histocompatibility complex; scFv, single-chain variable fragment; TGF, transforming growth factor; TNF, tumor necrosis factor.
